# Supplementary material for: HMGA2 Overexpression in Papillary Thyroid Cancer Promotes Thyroid Cell Dedifferentiation and Invasion, and These Effects Are Counteracted by Suramin
Source: Int J Mol Sci. 2025 Feb 14;26(4):1643. doi: 10.3390/ijms26041643 (PMC11854921; doi:10.3390/ijms26041643)
Supplement: Supplementary file 1 [file ijms-26-01643-s001.zip › ijms-3383629-supplementary.pdf]

Figure S1: (a) Assessment of SMAD2 protein phosphorylation levels by Western blot in TPC-1 cells treated with different concentrations of TGFβ (1, 10, 20, 100 ng/mL) for different durations (30 minutes, 1h, 24h; left panel) and with 10 ng/mL TFGβ for 1h and 72h (right panel) (C- : control untreated cells). **(b)** Schematic diagram showing the transfection and treatment protocol for TPC-1 and BCPAP cells (NC : negative control). (c) Analysis of the mRNA expression of SOX4, SNAI1 and SNAI2 by RT-qPCR in TPC-1 cells after treatment with TGFβ (10 ng/mL) for 1h (n=3) and 24h (n=4). An expression ratio for each mRNA was performed between TGFβ-treated and untreated TPC-1 cells (Fold change TGFβ/-TGFβ). ***P<0.001 vs. cells not treated with TGFβ.

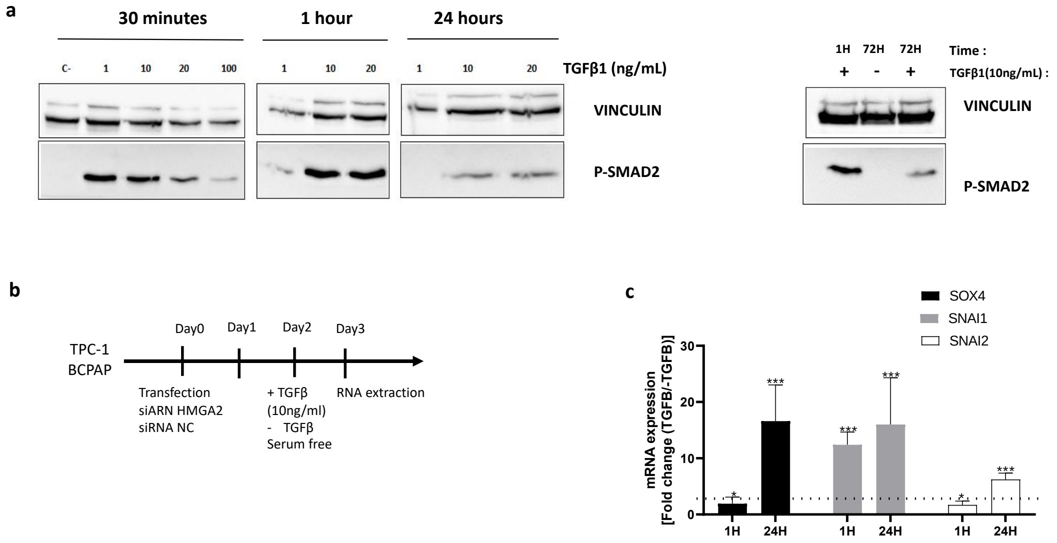

Figure S2 : HMGA2 protein expression is negatively correlated with TG protein expression in human PTC at cellular resolution level. Immunostaining of HMGA2 (yellow) and TG (green) on two adjacent sections of 2 PTC reveals the presence of HMGA2+/TG- and HMGA2-/TG+ cells. Epithelial cells were labeled with anti-CK8 antibody (red) and nuclei with DAPI (blue).

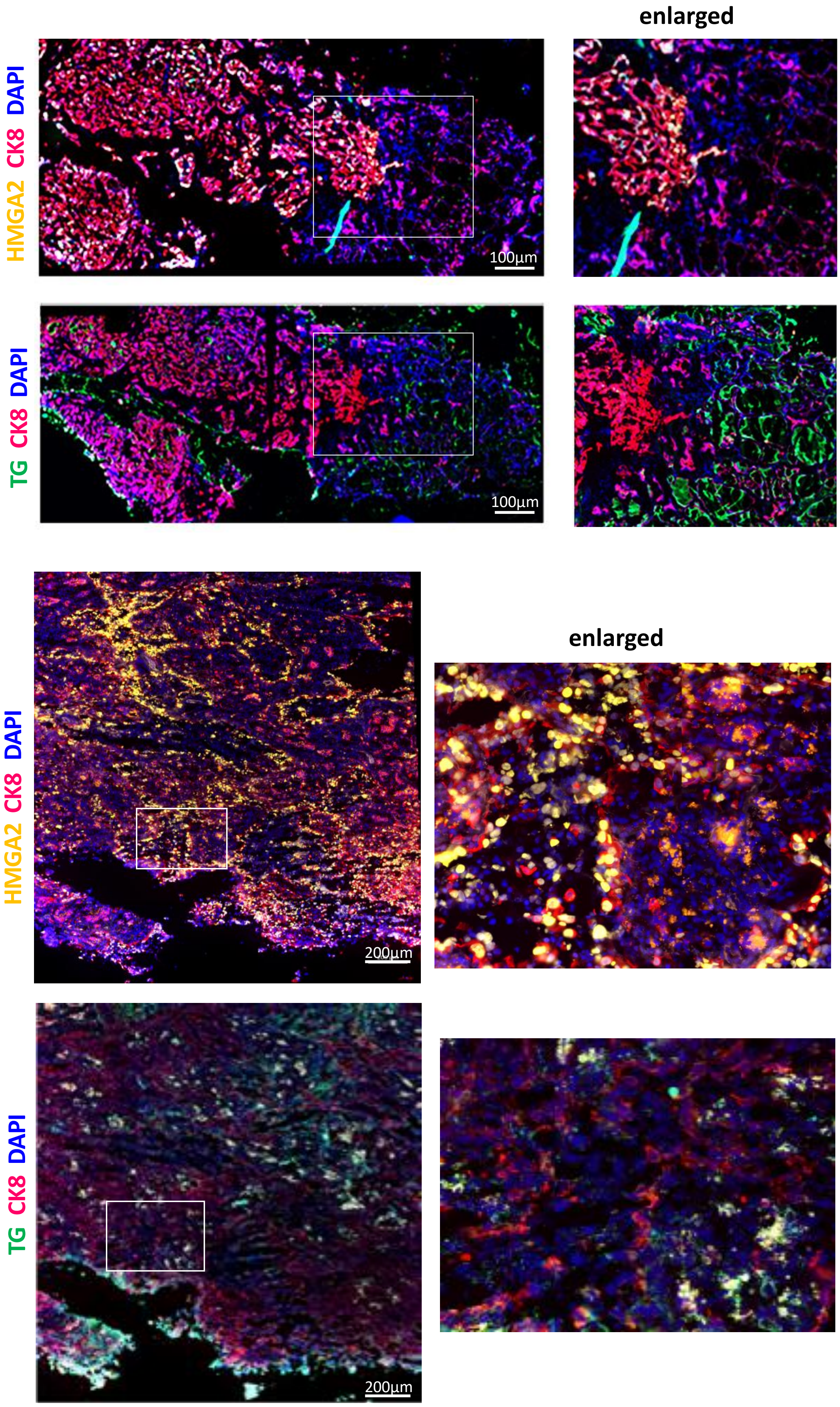

Supplementary Table 1. Clinicopathological features of PTC patients. All patients were tested $BRAF^{V600E}$ negative, except patient 2.

Patient ID	Diagnosis	Gender	Age	TNM
1	PTC, classic	F	45	pT1bN1a
2	PTC, classic	F	76	pT1bNx
3	PTC, follicular	M	61	pT1bNx
4	variant	F	35	pT1b(m)N0
5	PTC, classic	F	28	pT3N1b
6	PTC, classic	F	32	pT1N1b
7	PTC, classic	F	30	pT1b(m)
8	PTC, classic	F	51	pT1N0
9	PTC, classic	F	68	pT3N0
10	PTC, classic	F	23	pT2N0
11	PTC, follicular	M	14	pT3(M)N1b
	variant			
	PTC, diffuse			
	sclerosing variant			

Supplementary Table 2. Primer sequences list. All sequences should be read 5′ to 3′.

Human Gene ID	Primer forward	Primer reverse
SNAI1	CGAGTGGTTCTTCTGCGCTA	CTGCTGGAAGGTAAACTCTGGA
SNAI2	TCGGACCCACACATTACCTT	TTGGAGCAGTTTTTGCACTG
ZEB1	CTACACCGCCCAAAAAGAAA	AAGCGCTTTCCACATTTGTC
CDH1	GCTGAGCTGGACAGGGAGGA	ATGGGGGCGTTGTCATTCA
SOX4	GCTGGAAGCTGCTCAAAGAC	ACCGACCTTGTCTCCCTTCT
TGFBR2	TGCAAGATACATGGCTCCAG	CAACACGTTGTCCTTCATGC
HMGA2	AGTCCCTCTAAAGCAGCTCAAAAG	CCATTTCCTAGGTCTGCCTC
TG	GAGAAGAGCCTGTCGCTGAAA	CAGCTCACTGAACTCCTTGT
TPO	TTCCAGGAGCACCCCGAC	AGAACTCCCTCCACTCATTGTAACCT
PAX8	CTCACCTTCGCCATAAAGC	GCATGGGGAAAGGCATTGAA
NIS	ATCGCTATGGCCTCAAGTTCC	GCCGAGGTTTGATGAGGTCTT

Supplementary Table 3. Primer sequences list. All sequences should be read 5′ to 3′.

Mouse Gene ID	Primer forward	Primer reverse
HMGA2	ACATCAGCCCAGGGACAAC	TTCTGGGCTGCTTTAGAGGG
NIS	AGCTGCCAACACTTCCAGAG	GATGAGAGCACCACAAAGCA
TG	GTCCAATGCCAAAATGATGGTC	GAGAGCATCGGTGCTGTTAAT
TPO	ACAGTCACAGTTCTCCACGGATG	ATCTCTATTGTTGCACGCCCC
TSHR	GTCTGCCCAATATTTCCAGGATCTA	GCTCTGTCAAGGCATCAGGGT
NKX2.1	GGGCTCAAGCGCATCTCA	GGCGCCATGTCTTGTTCT
PAX8	CAGCCTGCTGAGTTCTCCAT	CTGTCTCAGGCCAAGTCCTC
TBP	TGTACCGCAGCTTCAAAATATTGTAT	AAATCAACGCAGTTGTCCGTG
HPRT	GCTACTGTAATGATCAGTCAACGGG	AAGCTTGCAACCTTAACCATTTGG
